# Genotype by *Phytophthora colocasiae* isolate interaction in breeding for resistance to taro [*Colocasia esculenta* var *esculenta* (L.) Schott] leaf blight disease in Ghana

**DOI:** 10.1016/j.heliyon.2023.e16350

**Published:** 2023-05-25

**Authors:** Esther Fobi Donkor, Daniel Nyadanu, Richard Akromah, Kingsley Osei

**Affiliations:** a University of Energy and Natural Resources, Department of Horticulture and Crop Production, Dormaa - Ahenkro Campus, Sunyani, Ghana; b Cocoa Research Institute, Plant Breeding Division, Tafo, Ghana; c Kwame Nkrumah University of Science and Technology, Kumasi, Department of Crops and Soil Sciences, Ghana; d Crop Research Institute of Council for Scientific and Industrial Research, Plant Pathology Department, Fumesua, Ghana

**Keywords:** *Colocasia esculenta*, *Phytophthora* leaf blight disease, Genotype by isolate interaction, Taro

## Abstract

Two experiments were undertaken to evaluate the resistance of 5 parents and 20 hybrids of dasheen taro (n = 25) developed through the diallel method to *Phytophthora* leaf blight disease which is also known as taro leaf blight disease (TLBD). The field experiment which was laid out in Randomized Complete Block Design with 3 replications assessed the disease incidence (%) and disease severity index (DSI%) among the genotypes planted in three (3) agro-ecological zones in Ghana. In the laboratory the parents and hybrids were inoculated with three (3) *Phytophthora colocasiae* isolates from Dormaa Ahenekro, Tano Dumasi and Bunso in the Bono, Ashanti and Eastern regions of Ghana respectively and the diameter of lesions formed on the leaves after five days of inoculation were measured. Significant differences (p < 0.05) among the parents and hybrids for resistance to the *Phytophthora colocasiae* isolates, TLBD incidence (%) and DSI (%) in the field were observed making room for selection for further breeding for resistant varieties of dasheen taro. The isolate effect and genotype by isolate interaction effect were not significant (p > 0.05) indicating the similarity of the isolates’ virulence and pathogenicity and therefore any of the isolates identified can be used to screen for resistance to TLBD in Ghana. Hybrids BL/SM/115 × BL/SM/10, BL/SM/115 × CE/MAL/32, CE/MAL/32 × BL/SM/10, CE/MAL/32 × CE/IND/16 and CE/IND/16 × BL/SM/115 and parent CE/IND/16 were observed to have no disease incidence with corresponding zero DSI (%) making them highly resistant and therefore can further be field evaluated and be recommended to farmers for cultivation. The highly significant and positive predictive value for TLBD incidence and DSI (%) based on the lesion area on leaf discs suggests that resistant genotypes of TLBD can be selected at the laboratory without spending long periods of time and money for evaluating genotypes in the field.

## 1 Introduction

Taro [*Colocasia esculenta* var. *esculenta* (L.) Schott] is among the widely cultivated root crops in Asia and Africa. It has other names such as kalo, dasheen, elephant's ear, gabi, binata, callaloo, eddo, eddy root and swamp taro [1,2]. It is a perennial crop which belongs to the genus *Colocasia* in the Araceae family [3]. Taro is an important staple crop for several small-scale farmers and is widely grown throughout Asia and the Pacific, the Americas and Africa [4]. It is consumed primarily for its starchy corm and leaves [5,6]. The corms are used as a source of carbohydrates, the leaves are consumed as vegetable, the petioles and flowers are also utilized as vegetables in certain parts of the world [7].

Currently the production of taro is threatened by the attack of many diseases and pests globally [8]. Diseases cause severe yield reductions and total plant death [9]. *Phytophthora* leaf blight disease also known as taro leaf blight disease (TLBD) is a very devastating disease which can cause up to 50% losses in corm yield and 95% losses in leaf yield [10,11]. The disease can defoliate a susceptible plant within 10 days during an epidemic. This causes reduction in carbohydrate production by photosynthesis and a reduction in corm yield [8]. It is a fungal disease caused by oomycete *Phytophthora colocasiae* which affects the leaves, petioles, and corms of the crop [12]. It can also cause a serious postharvest corm rot when corms are stored for a longer period usually after 7 days [13]. An outbreak of TLBD in some parts of the world caused a permanent shift from taro to sweet potato and cassava consumption and production, causing losses in millions of dollars in terms of export [14]. [15] was the first person to study the leaf blight disease of taro in Java and was also responsible for naming the causal pathogen, however there is limited information on the area of origin of the disease [16]. suggested that the disease might have originated from Asia as it is the center of origin for taro [17]. first reported the disease in India and currently has spread to most parts of the world including Africa and the Pacific. A major symptom of the disease is the presence of water-soaked lesions which occur initially as small dark spots on the upper surface of the leaves [9].

Omane [18] first reported the incidence of TLBD in Ghana after similar symptoms and yield loss of taro has been reported in other parts of West Africa. In 2009, there was an outbreak of TLBD in taro farms in the Eastern Region of Ghana and by the end of 2010 it had spread to other taro growing regions in the country making farmers to shift to the growing of rice and sugar cane [19]. Several management strategies have been employed against TLBD. The use of fungicide, biological and cultural practices have been effective, however the use of resistant varieties offers the most sustainable management strategy against TLB since it is extremely cost-effective and environmentally safe [11,20]. Availability of genetic resources and the resistance they confer is essential in breeding for resistance to TLBD. Breeding of resistant varieties of taro has been given a worldwide collaborative attention as the disease threatens the food security of poor and small holder farmers and also puts economies at risk [21]. This is evident in the activities of the International Network for Edible Aroids (INEA), The Centre for Pacific Crops and Trees (CePaCT), Taro Genetic Resources: Conservation and Utilization Network (TaroGen), Taro Network for South-east Asia and Oceania (TANSAO) which collected and shared genetic resources used to breed for resistance against TLB of which Ghana has been a beneficiary of these genetic resources [22,23].

The inheritance of resistance to TLBD falls under horizontal resistance category and it is polygenic and additively inherited making it more durable [24]. Information on the plant – pathogen interaction is very important for the breeding of resistant varieties. For TLBD, genotypes which show some form of resistance across environments are more desirable. Also, the genotype – pathogen interaction should be negligible [25]. The main objective of the study was therefore to evaluate five parents and 20 single cross hybrids of dasheen taro for resistance to TLBD.

## 2 Materials and methods

### 2.1 Planting materials

The five parents used in the study were selected based on performance from 35 accessions of dasheen taro evaluated for yield, nutrient content and resistant to TLBD. Parents BL/SM/10, CE/MAL/32, CE/IND/16 and BL/SM/151 which are exotic accessions from Samoa, Malaysia, Indonesia and Samoa respectively observed to be high yielding, TLBD resistance and high in nutrient contents and KAO 19 which is a local (from Ghana) accession was found to be moderately susceptible, high yielding and high in nutrient content during the preliminary studies of the work. The exotic parents were part of the germplasm distributed during 2004–2014 by Centre for Pacific Crops and Trees (CePaCT) as part of their “Fighting TLB Disease in Samoa and at Global Level Through Networking and Sharing Genetic Resources” program.

The parents were then hybridized in a 5 × 5 diallel mating design according to the hybridization protocol for taro from International Network for Edible Aroids (INEA) [23,26]. Gibberellic acid (GA_3_) was applied at a rate of 300 mg/L at 3 WAP (3 leaf stage) to induce and synchronize flowering of the genotypes (Amadi et al., 2015). The GA_3_ was mixed with a piece of detergent (key soap) to enable the solution stick to the surface of the taro leaves due to the waxy nature of the leaves. At 5 weeks after application of the GA_3,_ the genotypes started bearing flowers. Artificial pollination was done early in the morning from 6.00am to 9am when the flowers have started bringing out odors which is a sign of matured flowers. The male part of the flower was detached from the flower and the matured pollen brushed on the female part of the flowers. The crosses were then labelled and caped The emasculation process took 2 weeks as the flowers appeared in batches. The cap was removed after ten days of pollination to assess the success of the process. Successful berries which started forming clusters were then allowed to mature and dry on the plant. After 5 weeks of pollination, the dried berries were then harvested to the laboratory for seed extraction. The harvested berries were soaked in water over night. The berries were then washed in the water to remove the seeds which are the true taro seeds (TTS). A cheese cloth was used to strain the water from the seeds. The seeds were then dried at room temperature. True Taro Seeds (TTS) from the crosses were germinated in polybags in germinators. Due to the slow nature of multiplication of the seedlings, the micro propagation method was used to multiply the seedlings for the multi-locational trials. The F1s were selected based on their growth performance and its ability to produce more tillers for the multi-location trial.

### 2.2 Evaluation of genotypes for resistance to TLBD

**Fig. 1:**
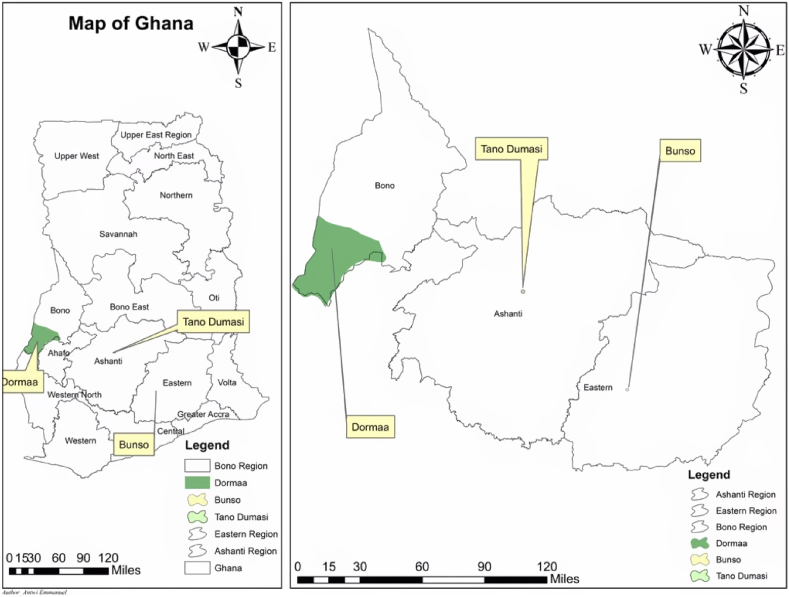
Map of Ghana showing the research locations.

Five parents and 20 single cross hybrids from the 5 × 5 complete diallel mating design, produced during the previous year, were planted using randomized complete block design (RCBD) with three replications in three locations from April to December. The three locations were; research field of School of Agriculture and Technology, University of Energy and Natural resources, Dormaa – Ahenkro (longitude 2°52.301′W, latitude 7°16.490′N and altitude 271 m) in the Bono Region, Plant Genetic Resources Research Institute (PGRI) of Council for Scientific and Industrial Research (CSRI), research field at Bunso (longitude 0°27.634′W, latitude 6°17.715′N and altitude 208.4 m) in the Eastern region and Ministry of Fisheries and Aqua Culture research site at Tano Dumasi (longitude1°30.2360′W, latitude 6°53.2530′N and altitude 277.1 m) in the Sekyere South District of Ashanti Region of Ghana. Table 1 shows the environmental conditions at the research locations during the research period and Fig. 1 is the map of Ghana showing the research locations. Six (6) plants were planted per plot for each genotype at a recommended planting distance of 1 m inter row and 0.5 m within row. Standard agronomic practices were followed in all the environments [27]. Well decomposed poultry manure was applied at a rate of 20 g per plant at 2 Weeks After Planting (WAP) to boost the growth of the plants. Table 2 shows the names details and status of parents and hybrids used in the research.

**Table 1:** Environmental conditions at the research locations during the research period.

Research location	Agro ecological zone	AVG. TEMP. (°C)	AVG. Rainfall (mm)	Soil type
Dormah Ahenkro	Forest Transition	25.7 °C	86.6	Loamy
Tano Dumasi	Forest Savannah Transition	21.5 °C	131.6	Clay Loam
Bunso	Semi Deciduous Forest	26.2 °C	117.1	Sandy Loam

AVG.TEMP = Average temperature, AVG. Rainfall = Average Rainfall.

**Table 2:** Name, details and status of parents and hybrids used in the research.

Genotype Name	Details	Status	
KAO19	P1	Parent	
BL/SM/10	P2	Parent	
BL/SM/115	P3	Parent	
CE/MAL/32	P4	Parent	
CE/IND/16	P5	Parent	
KAO19 × BL/SM/10	P1 × P2	Hybrid	
KAO19 × BL/SM/115	P1 × P3	Hybrid	
KAO19 × CE/MAL/32	P1 × P4	Hybrid	
KAO19 × CE/IND/16	P1 × P5	Hybrid	
BL/SM/10 × KAO19	P2 × P1	Hybrid	
BL/SM/10 × BL/SM/115	P2 × P3	Hybrid	
BL/SM/10 × CE/MAL/32	P2 × P4	Hybrid	
BL/SM/10 × CE/IND/16	P2 × P5	Hybrid	
BL/SM/115 × KAO19	P3 × P1	Hybrid	
BL/SM/115 × BL/SM/10	P3 × P2	Hybrid	
BL/SM/115 × CE/MAL/32	P3 × P4	Hybrid	
BL/SM/115 × CE/IND/16	P3 × P5	Hybrid	
CE/MAL/32 × KAO19	P4 × P1	Hybrid	
CE/MAL/32 × BL/SM/10	P4 × P2	Hybrid	
CE/MAL/32 × BL/SM/115	P4 × P3	Hybrid	
CE/MAL/32 × CE/IND/16	P4 × P5	Hybrid	
CE/IND/16 × KAO19	P5 × P1	Hybrid	
CE/IND/16 × BL/SM/10	P5 × P2	Hybrid	
CE/IND/16 × BL/SM/115	P5 × P3	Hybrid	
CE/IND/16 × CE/MAL/32	P5 × P4	Hybrid	

### 2.3 Inoculation of leaves of genotypes

*P. colocasiae* isolates isolated from infected leaves were used for the inoculation of the leaves. The diseased leaves were sampled from the three research locations that is Dormaa Ahenkro, Tano Dumasi and Bunsu. The *P. colocasiae* isolated from the diseased leaves were grown on Potato Dextrose Agar (PDA) as described by Ref. [28]. The isolates were named with the names of the towns from which the diseased leaves were sampled. The isolation was done at the Pathology Laboratory of Council for Scientific and Industrial Research – Crop Research Institute (CSIR- CRI), Fumesua, Ghana. The isolates were kept at 18 °C in a refrigerator and used when needed. Two weeks old young healthy and completely opened leaves were harvested from each genotype and sent to the pathology laboratory for inoculation with the *P. colocasiae* isolates using the leaf disc method [[28], [29], [30]]. Eight (8) cm diameter leaf disc was cut from the harvested leaves. The leaf disc was washed in water, then in bleached (0.5% concentration of Sodium hypochlorite) and then washed in water to sterilize the leaf surface. Filter paper was then wetted with sterile water and placed in Petri dish. The leaf disc was placed on the wet filter paper in the Petri dish. The Petri dish was covered and labelled appropriately. Two (2) weeks old culture of active growing isolates of *P. colocasiae* were used to inoculate the leaf disc in the Petri dish of all the genotypes using 3 mm agar plugs of the isolates. The inoculated leaf discs were kept at the incubation chamber at 28 °C for five days and arranged on the laboratory bench using Completely Randomized Design (CRD) with three replications. The two diameters (diameter 1 (D_1)_ and diameter 2 (D_2)_) of lesions that developed on the leaf discs after infection by isolates of *P. colocasiae* were measured and the area calculated as

Area of lesions = D_1_ × D_2_ (cm^2^)

where, D_1_ = diameter 1of the lesion and D_2_ = Diameter 2 of the lesion.

The process was followed for all the isolates from the three research locations for all the genotypes.

### 2.4 Evaluation of genotypes for disease incidence (%) and DSI (%)

At eight (8) Weeks After Planting (WAP) the research fields at each location was assessed for the incidence and DSI (%) of taro leaf blight disease among the parents and hybrids as described by Refs. [31,32] respectively. This was done by visual assessment.

$$Disease\;Incidence\left(\%\right)=\frac{number\;of\;infected\;plants}{total\;number\;of\;plants/plot}\times 100$$

$$Disease\;Severity\;Index\;\left(\%\right)=\frac{\left[sum\;\left(class\;frequency\times score\;of\;rating\;class\right)\right]}{\left[\left(total\;number\;of\;plants\right)\times \left(maximal\;disease\;index\right)\right]}\times 100\text{.}$$

The severity scale developed by [33] Omeje et al. (2015) was used to group the genotypes from highly resistant to susceptible. 0 = score <1 (highly resistant); 1 = score 1–25 (resistant); 2 = score 26–50 (moderately resistant); 3 = 51–75 (moderately susceptible); 4 = Score >75 (susceptible).

## 3 Statistical analysis

The analysis of variance (ANOVA) was conducted for the TLBD Lesion area (cm^2^), incidence (%) and DSI (%) using Statistical Tool for Agricultural Research [34] (STAR 2014) version 2.0.1 to determine the significance of the genotypes across the isolates. The means were separated using Least Significant Difference (LSD) at 5% level of significance. The data collected for disease incidence and DSI were transformed using the arc sine (angular) transformation before analysis of variance. AMMI analysis was also conducted to determine most important factor contributing to the total variation among the genotypes for disease incidence and DSI (%).

## 4 Results

### 4.1 Differences in lesion area among taro genotypes for resistance to *P. colocasiae* isolates

**Fig. 2:**
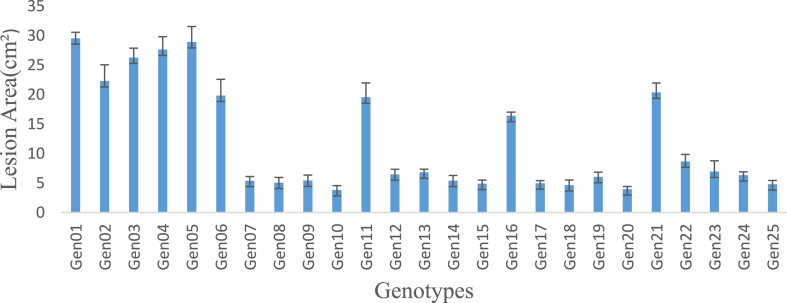
Genotypic differences in lesion size after inoculation of leaf discs with *P. colocasiae and* incubated for five days.

The ANOVA revealed significant differences (P < 0.05) among the genotypes for resistance to the *P. colocasiae* isolates. The mean lesion area was 12. 24 cm^2^ with hybrid BL/SM/10 × KAO 19 recording the least lesion area of 3.82 cm^2^ and parent KAO 19 recording the highest lesion area of 29.54 cm^2^. All the genotypes used as parents expressed above 20 cm^2^ lesion area while all the hybrids exhibited a lesion area below 10 cm^2^ except for hybrids KAO19 × BL/SM/10, BL/SM/10 × BL/SM/115, BL/SM/115 × CE/MAL/32 and CE/MAL/32 × CE/IND/16 which had above 10 cm^2^ lesion area. Fig. 2 shows the genotypic differences in lesion area on leaves of the various taro genotypes after inoculation with *P. colocasiae*.

The analysis also revealed significant differences (P < 0.001) among the genotypes for resistance to the *P. colocasiae* isolates. The isolate effect and genotype by isolate interaction were however not significant (P > 0.05) (Table 3).

**Table 3:** ANOVA parameters for main effects and interaction of genotypes and isolates of *Phytophthora colocasiae*.

Source of variation	d.f.	s.s.	m.s.	F pr.
Replication	2	91.3	45.65	
Genotype	24	18322.18	763.42	<.001
Isolate	2	7.02	3.51	0.822
Genotype × Isolate	48	959.4	19.99	0.301
Residual	148	2643.22	17.86	
Total	224	22023.12		

d.f*.* = degrees of freedom.

m.s = mean of squares.

s.s = sum of squares.

F pr. = F probability values.

### 4.2 Response of the genotypes to infection of the isolates of P. colocasiae

Tano Dumasi isolate caused the least mean lesion area of 11. 79 cm^2^ while isolates from Dormaa Ahenkro caused the highest lesion area of 12.20 cm^2^ (Table 4). All the parents, recorded high lesion area for all the isolates, while most of the hybrids recorded below 10 cm^2^ for all the three isolates except for KAO19 × BL/SM/10, BL/SM/10 × BL/SM/115, BL/SM/115 × CE/MAL/32, CE/MAL/32 × CINDY/16 which recorded above 10 cm^2^ lesion area for all the isolates.

**Table 4:** Mean lesion area caused by the three P. colocasiae isolates among the parents and single cross hybrids of taro.

GENOTYPE	Dormah Ahenkro Isolate *(cm*^*3*^)	Bunso Isolate *(cm*^*3*^)	Tano Dumasi Isolate *(cm*^*3*^)
KAO19	29.13	29.78	29.69
BL/SM/10	19.96	19.4	27.52
BL/SM/115	22.04	27.29	29.51
CE/MAL/32	25.85	29.89	27.15
CE/IND/16	23.03	33.07	30.61
KAO19 × BL/SM/10	26.41	18.62	14.48
KAO19 × BL/SM/115	6.27	5.55	4.3
KAO19 × CE/MAL/32	6.84	4.08	4.23
KAO19 × CE/IND/16	5.7	4.77	5.81
BL/SM/10 × KAO19	5.24	3.73	2.48
BL/SM/10 × BL/SM/115	23.55	18.35	16.73
BL/SM/10 × CE/MAL/32	4.66	9.17	5.54
BL/SM/10 × CE/IND/16	7.38	7.03	5.95
BL/SM/115 × KAO19	6.96	2.94	6.27
BL/SM/115 × BL/SM/10	4.99	5.8	3.87
BL/SM/115 × CE/MAL/32	17.79	16.28	16.16
BL/SM/115 × CE/IND/16	4.75	5.64	4.51
CE/MAL/32 × KAO19	2.83	4.95	6.15
CE/MAL/32 × BL/SM/10	6.47	7.88	3.77
CE/MAL/32 × BL/SM/115	3.48	4.67	3.72
CE/MAL/32 × CINDY/16	22.37	20.75	17.92
CE/IND/16 × KAO19	11.01	6.71	8.27
CE/IND/16 × BL/SM/10	6.8	4.61	9.46
CE/IND/16 × BL/SM/115	5.32	6.54	7.1
CE/IND/16 × CE/MAL/32	6.21	4.93	3.47
**Mean**	**12.2**	**12.1**	**11.79**

### 4.3 Response of the taro genotypes to natural field infection of *P. colocasiae*

Table 5 shows the mean square of TLBD incidence and DSI (%) for taro genotypes across the three environments. The genotypes differed significantly (P < 0.001) for TLBD incidence and DSI (%). The environments and the genotype by environment interaction effects were also highly significant (P < 0.001).

**Table 5:** Mean square of TLBD incidence and DSI (%) for taro genotypes across three environments.

Source of variation	df	Disease incidence (%)	DSI (%)
Replication	2	16.54	0.1733
Environment	2	12592.87***	49.1633***
Genotype	24	1612.51***	7.1169***
GEI	48	501.84***	1.8612***
Residual	148	37.2	0.2893
Total	224		

GEI= Genotype by Environment Interaction.

The AMMI (Additive Main effect and Multiplicative Interaction Model) analysis revealed highly significant (P < 0.001) differences for genotype, environment and GEI effect for TLBD incidence and DSI (%) (Table 6). Genotype effect contributed the highest proportion of the total variation followed by environment effect. GEI contributed the least proportion of the total variation for TLBD incidence and DSI (%) severity. All the two IPCAs (Interaction Principal Component axis) were highly significant (P < 0.001).

**Table 6:** AMMI analysis of variance for TLBD incidence and DSI (%) among taro genotypes evaluated across 3 environments.

		Disease Incidence (%)			DSI (%)		
Source	Df	MS	% TSS	%GEI	MS	%TSS	%GEI
Total	224	417			1.793		
Genotypes	24	1613***	41.39		7.117***	42.53	
Environments	2	12593***	26.93		49.163***	24.43	
Block	6	10			0.149		
GEI	48	502***	25.76		1.861***	22.23	
IPCA1	25	625***		64.88	2.495***		69.88
IPCA2	23	368***		35.12	1.172***		30.12
Residuals	0						
error	144	38			0.294		

%TSS = Total sum of squares.

IPCA 1 = Interaction Principal Component axis 1.

IPCA2 = Interaction Principal Component axis 2.

The Mean disease incidence and DSI (%) among the genotypes across the environments is presented in Table 7. The incidence and DSI (%) among the genotypes ranged from 0 to 49.44% and 0–32.78% with a mean incidence and DSI (%) of 13.60% and 9.60% respectively. Hybrids BL/SM/115 × BL/SM/10, BL/SM/115 × CE/MAL/32, CE/MAL/32 × BL/SM/10, CE/MAL/32 × CE/IND/16 and CE/IND/16 × BL/SM/115 and parent CE/IND/16 were observed to have no disease incidence with corresponding zero DSI (%) for TLBD on the field. Parent KAO 19, a local accession, had the highest disease incidence of 49.44% and DSI (%) of 32.78%. All the parents recorded high disease incidence except for CE/IND/16. All the hybrids with parent KAO 19 as a parent recorded high disease incidence with corresponding high DSI (%) (Table 7). The high cv (%) for the disease incidence and DSI (%) could be due to the high level of variation among the 3 environments used. This is clearly shown by the variations in weather data of the three locations (Table 1). The diseases incidence and DSI (%) of the genotypes in the various environments has been presented as a supplementary table (Appendix 1 and 2).

**Table 7:** Disease incidence and DSI (%) among parents and single cross hybrids of taro (dasheen).

Genotype	Incidence (%)	DSI (%)
KAO19	49.44 **(1.1.57)**	32.78 **(1.57)**
BL/SM/10	34.44 **(0.99)**	22.22 **(0.97)**
BL/SM/115	11.67 **(0.51)**	8.33 **(0.53)**
CE/MAL/32	11.56 **(0.51**	14.44 **(0.73)**
CE/IND/16	0.00 **(0)**	0.00 **(0)**
KAO19 × BL/SM/10	41.33**(1.15)**	26.11**(1.10)**
KAO19 × BL/SM/115	12.22 **(0.52)**	12.22 **(0.66)**
KAO19 × CE/MAL/32	16.67**(0.62)**	10.56 **(0.60)**
KAO19 × CE/IND/16	19.44**(0.68)**	11.11 **(0.62)**
BL/SM/10 × KAO19	9.33 **(0.45)**	8.89 **(0.55)**
BL/SM/10 × BL/SM/115	35.78 **(1.02)**	26.11**(1.10)**
BL/SM/10 × CE/MAL/32	8.22 **(0.42)**	8.89 **(0.55)**
BL/SM/10 × CE/IND/16	10.00 **(0.47)**	6.11 **(0.45)**
BL/SM/115 × KAO19	13.89 **(0.56)**	7.78 **(0.51)**
BL/SM/115 × BL/SM/10	0.00 **(0)**	0.00 **(0)**
BL/SM/115 × CE/MAL/32	0.00 **(0)**	0.00 **(0)**
BL/SM/115 × CE/IND/16	11.11 **(0.49)**	8.33 **(0.53)**
CE/MAL/32 × KAO19	11.67 **(0.51)**	6.67 **(0.47)**
CE/MAL/32 × BL/SM/10	0.00 **(0)**	0.00 **(0)**
CE/MAL/32 × BL/SM/115	9.44 **(0.45)**	8.33 **(0.53)**
CE/MAL/32 × CIN/IND//16	0.00 **(0)**	0.00 **(0)**
CE/IND/16 × KAO19	17.22 **(0.63)**	10.56 **(0.60)**
CE/IND/16 × BL/SM/10	8.89 **(0.44)**	4.44 **(0.38)**
CE/IND/16 × BL/SM/115	0.00 **(0)**	0.00 **(0)**
CE/IND/16 × CE/MAL/32	7.78 **(0.41)**	6.11 **(0.45)**
Mean	13.60 (0.55)	9.60 (0.57)
cv (%)	24.80	26.00
lsd (5%)	5.68	5.01

*Transformed figures in parenthesis and bold.

cv (%) = coefficient of variation.

LSD = Least significant levels.

Based on the severity scale, 5 hybrids, that is, BL/SM/115 × BL/SM/10, BL/SM/115 × CE/MAL/32, CE/MAL/32 × BL/SM/10, CE/MAL/32 × CE/IND/16 and CE/IND/16 × BL/SM/115 and parent CE/IND/16 were found to be highly resistant and sixteen (16) genotypes which includes parents BL/SM/10 and BL/SM/115, were found to be resistant. Two hybrids KAO 19 × BL/SM/10 and BL/SM/10 × BL/SM/115 and parent KAO 19 (a local accession) were found to be moderately resistant. None of the genotype was susceptible (Table 8).

**Table 8:** Disease severity scale, number and names of genotypes for each scale.

Scales	Severity score range	Description	Number of genotypes	Genotypes
0	<1	Highly resistant	6	CE/IND/16, BL/SM/115 × BL/SM/10, BL/SM/115 × CE/MAL/32, CE/MAL/32 × BL/SM/10, CE/MAL/32 × CE/IND/16 and CE/IND/16 × BL/SM/115
1	1–25	resistant	16	BL/SM/10, BL/SM/115, CE/MAL/32, KAO19 × BL/SM/115, KAO 19 × CE/MAL/32, KAO 19 × CE/IND/16, BL/SM/10 × KAO 19, BL/SM/10 × CE/MAL/32, BL/SM/10 × CE/IND/16. BL/SM/115 × KAO 19, BL/SM/115 × CE/IND/16, CE/MAL/32 × KAO 19, CE/MAL/32 × BL/SM/115, CE/IND/16 × KAO 19, CE/IND/16 × BL/SM/10 and CE/IND/16 × CE/MAL/32
2	26–50	Moderately resistant	3	KAO 19, KAO 19 × BL/SM/10 and BL/SM/10 × BL/SM/115
3	51–75	Moderately susceptible	0	NIL
4	>75	Susceptible	0	NIL

4.4 Regression analysis between TLBD lesion area assessed in the laboratory and incidence and DSI (%) of natural infection of the disease in the field.

**Fig. 3:**
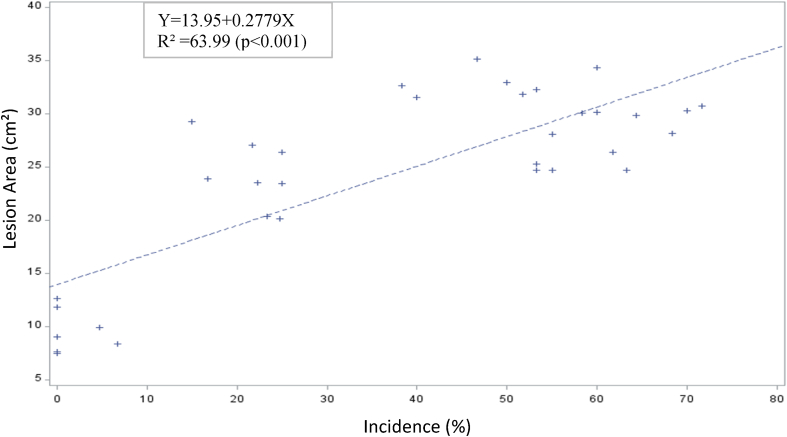
Reliability of prediction of TLBD incidence (%) on the field based on the lesion area on leaves inoculated with P. colocasiae inoculum in the laboratory.

**Fig. 4:**
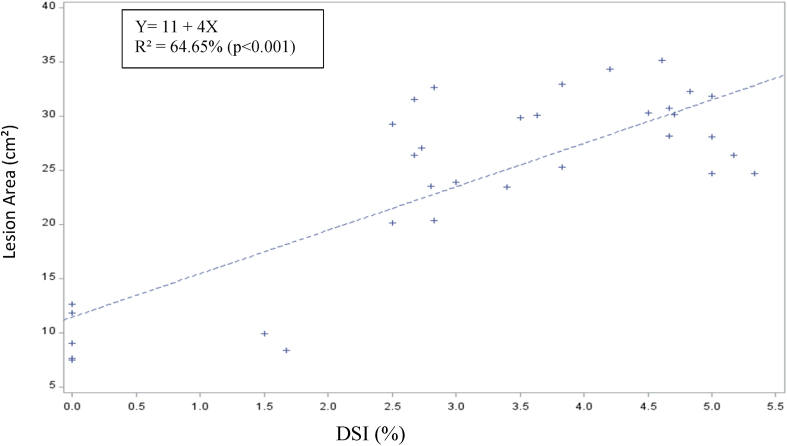
Reliability of prediction of TLBD DSI (%) on the field based on the lesion area on leaves inoculated with P. colocasiae inoculum in the laboratory.

The predictive value of TLBD lesion area (cm^2^) on disease incidence is high (63.99%) and highly significant (P < 0.001) (Fig. 3) and also high (64.65%) and highly significant (P < 0.001) for disease DSI (%) (Fig. 4). The intercepts for the models were positive for both traits.

## 5 Discussion

Host plant resistance is the best and reliable breeding method for controlling diseases [35,36]. Host plant resistance is therefore considered the most appropriate method for dealing with TLBD. The significant variability among the taro genotypes for the TLBD lesion area and incidence and DSI (%) of natural infection in the field (Table 1, Table 5) suggested a wide scope of selection for resistance to TLBD. This provides the opportunity for selection among the genotypes for further screening for resistance to TLBD in varietal development program of taro in Ghana. Similar results were reported in taro by Refs. [[37], [38], [39], [40]]. [41] also reported significant differences for resistance to late blight (*Phytophthora infestans)* in potatoes.

Genotype by isolate interaction (GII) is one of the hindrances in breeding for disease resistance in crops. Significant GII requires that the breeder breeds for resistance to the individual isolates of the pathogen identified. The non-significant difference (P > 0.05) among the isolates ability to cause lesions on the leaves and the GII (Table 3) reveals that the virulence and reaction of isolates of *P. colocasiae* to infection on the leaves of the taro genotypes is similar. This suggest that breeding for resistance to TLBD can be done using any of the isolates. However, using the most aggressive isolate would be more appropriate as it will help to identify more useful levels of resistance of the genotypes against the pathogen. This is confirmed in this study as genotypes which expressed high or low lesion area for one isolate, express the same for all the other isolates (Table 4). Therefore, a genotype which is resistant to one of the isolates will be resistant to other *P. colocasiae* isolates that will be identified. The lack of specificity between the *P. colocasiae* isolates and the taro genotypes maybe universal as the exotic genotypes which were selected for resistance to the TLBD in the Fighting TLBD in Samoa and at Global Level Through Networking and Sharing Genetic Resources Program” also showed resistance to the *P. colocasiae* isolates used in this research. This gives a justification of a pre-breeding program done in a well-resourced country to accumulate genes for resistance to TLBD and the genetic material distributed to other countries where the disease is devastating. These findings agree with the work of [42] who also reported non-significant (P > 0.05) GII for *P. colocasiae* in selection against TLBD. Similar findings were also reported by Refs. [25,43] in cocoa for resistance to *P. palmivora* isolates. These findings however contradict the results of [29] who reported significant (P < 0.05) GII for four *P. colocasiae* isolates.

Hybrids BL/SM/10 × KAO 19, BL/SM/115 × BL/SM/10, BL/SM/115 × CE/IND/16, CE/MAL/32 × KAO 19, CE/MAL/32 × BL/SM/115, CE/IND/16 × CE/MAL/32 and parent CE/IND/16 which recorded low lesion area and no disease incidence and therefore grouped as resistant can be included in breeding programs for resistance to TLBD in Ghana. The low disease incidence of the parents except KAO 19, a local accession, was due to the fact that the introduced accessions were included in the ongoing worldwide breeding program against the TLBD by Centre for Pacific Crops and Trees (CePaCT) as part of their “Fighting TLBD in Samoa and at Global Level Through Networking and Sharing Genetic Resources Program” and therefore selection against TLBD was carried out earlier for these genotypes. The local accession however is a landrace and have not seen any selection against TLBD [19].also reported low incidence and severity of TLBD on research fields planted with accessions from Samoa and Vanuatu. The grouping of some of the hybrids from the local accessions as resistant according to the severity scale (Table 8) provides hope of including a local accession in breeding program to develop resistant varieties of taro in Ghana which will be more adopted to the local environment.

The highly significant differences (P < 0.001) among the environments for TLBD incidence and DSI (%) (Table 5) suggest that environmental factors play a role in the incidence and DSI (%) of TLBD. The significant GEI implies that the genotypes performed differently in the three environments, indicating the need to evaluate the genotypes in different environments for selection of resistant genotypes. However, the higher contribution of genotype effect than environment and GEI to the total variation (Table 6) suggest that the differences in the genotypes are the most important factors to consider when selecting genotypes for resistance to TLBD and that phenotypic selection will be appropriate for the improvement of genotypes for resistance to TLBD. The least contribution of GEI to the total variation implies that the best discriminating environment can be used to evaluate the genotypes for resistance to TLBD [44]. reported similar results for cassava brown streak disease root necrosis [45]. however, reported higher environmental effect than genotype and GEI effect for resistance to sweet potato virus disease among sweet potato genotypes in Tanzania.

The highly significant and positive predictive value for TLBD incidence and DSI (%) based on the lesion area (Fig. 3, Fig. 4) suggests that resistant genotypes of TLBD can be selected at the laboratory without spending long periods of time and money evaluating genotypes in the field. Similar result has been reported by Ref. [46] for black pod disease in cocoa.

## 6 Conclusion

The study revealed high level of diversity among the genotypes for resistance to TLBD and non-significant (P > 0.05) genotype by isolate interaction for the *P. colocasiae* isolates used making room for breeding for resistance using any of the isolates identified in Ghana. Hybrids BL/SM/115 × BL/SM/10, BL/SM/115 × CE/MAL/32, CE/MAL/32 × BL/SM/10, CE/MAL/32 × CE/IND/16 and CE/IND/16 × BL/SM/115 which were highly resistant to the TLBD could be further evaluated for release to farmers.

## Author contribution statement

Esther Fobi Donkor: Conceived and designed the experiments; Performed the experiments; Analyzed and interpreted the data; Contributed reagents, materials, analysis tools or data; Wrote the paper.

Daniel Nyadanu: Conceived and designed the experiments; Wrote the paper.

Richard Akromah; Kingsley Osei: Contributed reagents, materials, analysis tools or data; Wrote the paper.

## Data availability statement

Data will be made available on request.

## Additional information

Supplementary content related to this article has been published online at [URL].

## Declaration of competing interest

The authors declare that they have no known competing financial interests or personal relationships that could have appeared to influence the work reported in this paper.
