# Supplementary material for: Genotype by Phytophthora colocasiae isolate interaction in breeding for resistance to taro [Colocasia esculenta var esculenta (L.) Schott] leaf blight disease in Ghana
Source: Heliyon. 2023 May 25;9(6):e16350. doi: 10.1016/j.heliyon.2023.e16350 (PMC10238886; doi:10.1016/j.heliyon.2023.e16350)
Supplement: Supplementary file 1 [file mmc1.docx]

|  |  | Disease incidence (%) | | |
| --- | --- | --- | --- | --- |
| S/n | Genotype | Environment 1 | Environment 2 | Environment 3 |
| 1 | KAO19 | 35.67 ±33.08 | 64.67 ±51.53 | 37.00 ±32.92 |
| 2 | BL/SM/10 | 0.00 ±2.59 | 51.67 ±38.53 | 0.00 ±4.08 |
| 3 | BL/SM/115 | 0.00 ±2.59 | 0.00 ±13.13 | 0.00 ±4.08 |
| 4 | CE/MAL/32 | 0.00 ±2.59 | 17.33 ±4.20 | 0.00 ±4.08 |
| 5 | CE/IND/16 | 0.00 ±2.59 | 0.00 ±13.13 | 0.00 ±4.08 |
| 6 | KAO19 $\times$ BL/SM/10 | 17.33 ±14.75 | 53.33 ±40.20 | 33.00±28.92 |
| 7 | KAO19 $\times$ BL/SM/115 | 0.00 ±2.59 | 18.33 ±5.20 | 0.00 ±4.08 |
| 8 | KAO19 $\times$ CE/MAL/32 | 0.00 ±2.59 | 50.00 ±36.87 | 0.00 ±4.08 |
| 9 | KAO19 $\times$ CE/IND/16 | 0.00 ±2.59 | 0.00 ±13.13 | 0.00 ±4.08 |
| 10 | BL/SM/10 $\times$ KAO19 | 0.00 ±2.59 | 14.00 ±0.87 | 0.00 ±4.08 |
| 11 | BL/SM/10 $\times$ BL/SM/115 | 11.67 ±9.08 | 45.00 ±31.87 | 32.00±27.92 |
| 12 | BL/SM/10 $\times$ CE/MAL/32 | 0.00 ±2.59 | 14.00 ±0.87 | 0.00 ±4.08 |
| 13 | BL/SM/10 $\times$ CE/IND/16 | 0.00 ±2.59 | 0.00 ±13.13 | 0.00 ±4.08 |
| 14 | BL/SM/115 $\times$ KAO19 | 0.00 ±2.59 | 0.00 ±13.13 | 0.00 ±4.08 |
| 15 | BL/SM/115 $\times$ BL/SM/10 | 0.00 ±2.59 | 0.00 ±13.13 | 0.00 ±4.08 |
| 16 | BL/SM/115 $\times$ CE/MAL/32 | 0.00 ±2.59 | 0.00 ±13.13 | 0.00 ±4.08 |
| 17 | BL/SM/115 $\times$ CE/IND/16 | 0.00 ±2.59 | 0.00 ±13.13 | 0.00 ±4.08 |
| 18 | CE/MAL/32 $\times$ KAO19 | 0.00 ±2.59 | 0.00 ±13.13 | 0.00 ±4.08 |
| 18 | CE/MAL/32 $\times$ BL/SM/10 | 0.00 ±2.59 | 0.00 ±13.13 | 0.00 ±4.08 |
| 20 | CE/MAL/32 $\times$ BL/SM/115 | 0.00 ±2.59 | 0.00 ±13.13 | 0.00 ± 4.08 |
| 21 | CE/MAL/32 $\times$ CE/IND/16 | 0.00 ±2.59 | 0.00 ±13.13 | 0.00 ±4.08 |
| 22 | CE/IND/16 $\times$ KAO19 | 0.00 ±2.59 | 0.00 ±13.13 | 0.00 ±4.08 |
| 23 | CE/IND/16 $\times$ BL/SM/10 | 0.00 ±2.59 | 0.00 ±13.13 | 0.00 ±4.08 |
| 24 | CE/IND/16 $\times$ BL/SM/115 | 0.00 ±2.59 | 0.00 ±13.13 | 0.00 ±4.08 |
| 25 | CE/IND/16 $\times$ CE/MAL/32 | 0.00 ±2.59 | 0.00 ±13.13 | 0.00 ±4.08 |
|  | Grand mean | 2.59 | 13.13 | 4.08 |
|  | CV | 22.56 | 21.5 | 35.0 |
|  | LSD | 9.57 | 4.62 | 2.34 |

Appendix 1: Disease incidence of the genotypes in the 3 research locations

Appendix 2: Disease severity of the genotypes in the 3 research locations

|  |  | Disease severity | | |
| --- | --- | --- | --- | --- |
| S/n | Genotype | Environment 1 | Environment 2 | Environment 3 |
| 1 | KAO19 | 2.50 ±2.26 | 3.67 ±2.77 | 2.83 ±2.53 |
| 2 | BL/SM/10 | 0.00 ±0.24 | 3.33 ±2.43 | 0.00 ±0.30 |
| 3 | BL/SM/115 | 0.00 ±0.24 | 0.00 ±0.90 | 0.00 ±0.30 |
| 4 | CE/MAL/32 | 0.00 ±0.24 | 2.17 ± 1.27 | 0.00 ±0.30 |
| 5 | CE/IND/16 | 0.00 ±0.24 | 0.00 ±0.90 | 0.00 ±0.30 |
| 6 | KAO19 $\times$ BL/SM/10 | 1.83 ±1.59 | 3.00 ±2.10 | 2.83 ±2.53 |
| 7 | KAO19 $\times$ BL/SM/115 | 0.00 ±0.24 | 1.83 ±0.93 | 0.00 ±0.30 |
| 8 | KAO19 $\times$ CE/MAL/32 | 0.00 ±0.24 | 3.17 ±2.27 | 0.00 ±0.30 |
| 9 | KAO19 $\times$ CE/IND/16 | 0.00 ±0.24 | 0.00 ±13.13 | 0.00 ±0.30 |
| 10 | BL/SM/10 $\times$ KAO19 | 0.00 ±0.24 | 1.17 ±0.27 | 0.00 ±0.30 |
| 11 | BL/SM/10 $\times$ BL/SM/115 | 1.67 ±1.43 | 2.83 ±1.93 | 1.83 ±1.53 |
| 12 | BL/SM/10 $\times$ CE/MAL/32 | 0.00 ±0.24 | 1.33 ±0.43 | 0.00 ±0.30 |
| 13 | BL/SM/10 $\times$ CE/IND/16 | 0.00 ±0.24 | 0.00 ±0.90 | 0.00 ±0.30 |
| 14 | BL/SM/115 $\times$ KAO19 | 0.00 ±0.24 | 0.00 ±0.90 | 0.00 ±0.30 |
| 15 | BL/SM/115 $\times$ BL/SM/10 | 0.00 ±0.24 | 0.00 ±0.90 | 0.00 ±0.30 |
| 16 | BL/SM/115 $\times$ CE/MAL/32 | 0.00 ±0.24 | 0.00 ±0.90 | 0.00 ±0.30 |
| 17 | BL/SM/115 $\times$ CE/IND/16 | 0.00 ±0.24 | 0.00 ±0.90 | 0.00 ±0.30 |
| 18 | CE/MAL/32 $\times$ KAO19 | 0.00 ±0.24 | 0.00 ±0.90 | 0.00 ±0.30 |
| 18 | CE/MAL/32 $\times$ BL/SM/10 | 0.00 ±0.24 | 0.00 ±0.90 | 0.00 ±0.30 |
| 20 | CE/MAL/32 $\times$ BL/SM/115 | 0.00 ±0.24 | 0.00 ±0.90 | 0.00 ± 0.30 |
| 21 | CE/MAL/32 $\times$ CE/IND/16 | 0.00 ±0.24 | 0.00 ±0.90 | 0.00 ±0.30 |
| 22 | CE/IND/16 $\times$ KAO19 | 0.00 ±0.24 | 0.00 ±0.90 | 0.00 ±0.30 |
| 23 | CE/IND/16 $\times$ BL/SM/10 | 0.00 ±0.24 | 0.00 ±0.90 | 0.00 ±0.30 |
| 24 | CE/IND/16 $\times$ BL/SM/115 | 0.00 ±0.24 | 0.00 ±0.90 | 0.00 ±0.30 |
| 25 | CE/IND/16 $\times$ CE/MAL/32 | 0.00 ±0.24 | 0.00 ±0.90 | 0.00 ±0.30 |
|  | Grand mean | 0.24 | 0.90 | 0.30 |
|  | CV | 54.00 | 65.00 | 81.60 |
|  | LSD | 0.61 | 0.89 | 0.40 |
